# Clinical characteristics of headaches in an urban Mennonite group in South Brazil

**DOI:** 10.1055/s-0043-1772603

**Published:** 2023-10-04

**Authors:** David Lemke Dück, Marco Antonio Takashi Utiumi, Angelica Beate Winter Boldt, Elcio Juliato Piovesan

**Affiliations:** 1 Universidade Federal do Paraná, Setor de Ciências da Saúde, Curitiba PR, Brazil.; 2 Clínica de Neurologia São José, Centro de Cefaleia, São José dos Pinhais PR, Brazil.; 3 Universidade Federal do Paraná, Departamento de Genética, Curitiba PR, Brazil.; 4 Universidade Federal do Paraná, Departamento de Clínica Médica, Curitiba PR, Brazil.

**Keywords:** Reproductive Isolation, Migraine Disorders, Urban Population, Headache, Isolamento Reprodutivo, Transtornos de Enxaqueca, População Urbana, Cefaleia

## Abstract

**Background** Genetic variants play a pathophysiological role in headaches, especially in migraine. The Mennonite group (MG) has been geographically and genetically isolated throughout its history, harboring a distinctive distribution of diseases.

**Objective** To determine the characteristics of headaches in a group with direct Mennonite ancestry contrasting with other urban community members (control group [CG]).

**Methods** Subjects with headaches were asked to complete a questionnaire covering: the type of headache, presence of aura, frequency and duration of attacks, pain location and severity, analgesic consumption, premonitory and postdromic manifestations, Depressive Thoughts Scale, Epworth Sleepiness Scale (ESS), General Anxiety Disorder-7, Patient Health Questionnaire-9 (PHQ-9), Migraine Disability Assessment, and Composite Autonomic System Score.

**Results** We included 103 participants (CG: 45, Mennonite group [MG]: 58). Migraine was the most common headache (CG: 91.1%; MG: 81.0%; *p* = 0.172), followed by tension-type headache (CG: 8.9%; MG: 15.5%; *p* = 0.381). Aura was identified by 44.4% and 39.7% in the CG and MG, respectively ( *p* = 0.689). The groups differed only concerning the frequency of retro-orbital pain (CG: 55.6%; MG: 32.8%; *p* = 0.027), PHQ-9 (CG: median 7, range 0 to 22; MG: median 5, range 0 to 19; *p* = 0.031) and ESS (CG: median 0, range 0 to 270; MG: median 0, range 0 to 108; *p* = 0.048) scores.

**Conclusion** There were no major differences in the prevalence and clinical characterization of headaches between the MG and the CG. However, the latter showed more diffuse pain, sleepiness, and depressive symptoms. Specific genetic or epigenetic variants in Mennonite descendants might account for these differences.

## Resumo

**Antecedentes** Variantes genéticas desempenham um papel fisiopatológico nas cefaleias, especialmente na migrânea. O grupo menonita (GM) tem estado geográfica e geneticamente isolado ao longo de sua história, abrigando uma distribuição distinta de doenças.

**Objetivo** Determinar as características das cefaleias em um grupo com ascendência menonita direta, comparando-as com as de outros membros da comunidade urbana (grupo controle [GC]).

**Métodos** Participantes com cefaleia foram convidados a preencher um questionário abrangendo: tipo de cefaleia; presença de aura; frequência e duração dos ataques; localização e gravidade da dor; consumo de analgésicos; manifestações premonitórias e posdrômicas; Escala de Pensamentos Depressivos; Escala de Sonolência de Epworth (ESS); Transtorno de Ansiedade Geral-7 (GAD-7); Questionário de Saúde do Paciente-9 (PHQ-9); Avaliação de Incapacidade da Migrânea (MIDAS) e Escore do Sistema Autônomo Composto (COMPASS-31).

**Resultados** Incluímos 103 participantes (GC: 45, GM: 58). A migrânea foi a cefaleia mais frequente (GC: 91,1%; GM: 81,0%; *p* = 0,172), seguida pela cefaleia tensional (GC: 8,9%; GM: 15,5%; *p* = 0,381). Aura foi identificada por 44,4% e 39,7% nos GC e GM, respectivamente ( *p* = 0,689). Os grupos diferiram apenas com relação à frequência de dor retro-orbitária (GC: 55,6%; GM: 32,8%; *p* = 0,027), PHQ-9 (GC: mediana 7, amplitude 0 a 22; GM: mediana 5, amplitude 0 a 19; *p* = 0,031) e ESS (GC: mediana 0, amplitude 0 a 270; GM: mediana 0, amplitude 0 a 108; *p* = 0,048).

**Conclusão** Não houve diferenças significativas na prevalência e caracterização clínica das cefaleias nos GM e GC. Entretanto, o último grupo mostrou mais dor difusa, sonolência e sintomas depressivos. Variantes genéticas ou epigenéticas específicas em descendentes de menonitas podem justificar tais diferenças.

## INTRODUCTION

The Mennonites are an Anabaptist group of Frisian/Flemish origin that originated after the Protestant Reform in the 16 ^th^ century. Advocating for a more radical reformation, including pacifism and the doctrine of adult baptism, the followers of the former priest Menno Simons were religiously and politically persecuted and thus ended up becoming an isolated population. 1 Genetic drift, reproductive isolation, and at least three bottleneck effects in this population are expected to increase the frequencies of homozygotes and rare alleles, changing the prevalence of chronic diseases. 1 2 3 4 An example of this would be the higher prevalence of dystonia in the Amish, also an Anabaptist population. 5

There are several different subgroups of Mennonites. The genetic epidemiology of complex and rare diseases and phenotypes have been described more extensively in the Amish and somewhat also in Canadian and Kansas Mennonites. 6 7 In contrast, South American Mennonites are still epidemiologically poorly known, despite some advances regarding their genetic heritage. 1 4 8

About three billion people have tension-type headache (TTH) or migraine (1.89 and 1.04 billion, respectively), the most common headaches in the general population. 9 Regarding prevalence, TTH is the third most frequent condition in the world and migraine is the sixth. 10 In Brazil, approximately 13% of the population suffers from TTH and 15.2% from migraine. 11 12 This prevalence could be even higher in some subpopulations depending on factors such as the female gender and depression. 13

Headaches are a public health concern. Tension-type headache caused 7.2 million YLDs (years of life lived with disability) and migraine 45.1 million YLDs. 9 While TTH tends to be more pervasive, migraine tends to be more severe and can progress through several stages. A premonitory phase could present with fatigue and stiff neck, among other symptoms, as the first manifestation. 14 Second, an aura phase can be reported by a third of patients. 15 Next, headache pain arises, accompanied by symptoms such as photophobia, phonophobia, osmophobia, allodynia, and vertigo. As the pain resolves, some symptoms persist in the final postdromic phase. 16

The exact molecular mechanisms responsible for migraine are still unclear. It has been suggested a role for neuropeptides such as calcitonin gene-related peptide (CGRP) 17 and pituitary adenylate cyclase-activating polypeptide 18 (PACAP-38) that cause vasodilation by the increase of cyclic adenosine monophosphate (cAMP) in the smooth muscle of vessels and are found at higher levels in the peripheral blood during the attack. Furthermore, CGRP infused intravenously triggers a delayed migraine-like headache in patients with migraine without aura (MO). 19

The phenotype of migraine might depend on genetic polymorphisms and epigenetic gene regulation. Migraine with aura (MA) and MO are associated with genetic variants that increase their risk. 20 This relationship is more robust in rarer forms of the disease, such as familial hemiplegic migraine, but is also found in MA and, to a lesser extent, in MO. Thus, both rare pathological mutations in genes encoding specific ion channels and common gene variants contribute to migraine. However, the latter is the main family aggregation factor of the disease. 21

Epigenetic processes could mediate our response to food, environment, and stress challenges, among other elements clinically known as trigger factors for migraine attacks. The CGRP gene ( *CALCA* ) expression can be modified in several ways (DNA methylation, histone modifications, and noncoding RNAs), and the CGRP might, in turn, trigger regulatory mechanisms in neuronal and glial cells. 22

In the present study, we sought to determine the characteristics of headaches from a semiological point of view in Mennonites, comparing them with Brazilian non-Mennonites, subject to a similar environment (epigenetic factors).

## METHODS

The present study was carried out within the genetic-epidemiological Mennogen project with the South-Brazilian Mennonite population. 2 In order to retrospectively and cross-sectionally evaluate the characteristics of headaches in this population, we publicized and explained the research goals in social and religious events. Individuals interested in participating in the survey received access to the questionnaires through a Google Forms platform link. Before answering the questions, all subjects were required to fill in an informed consent form. The study was approved by the Ethics Committee of the Health Sciences Sector of the UFPR (CAAE: 54385616.2.0000.0102).

We evaluated the following data:

- epidemiological information;
- headache characteristics;
- Depressive Thoughts Scale 23 (EPD);
- Epworth Sleepiness Scale 24 (ESS);
- General Anxiety Disorder-7 25 (GAD-7);
- Patient Health Questionnaire-9 26 (PHQ-9);
- Migraine Disability Assessment 27 (MIDAS);
- Composite Autonomic Symptom Score 28 (COMPASS 31).

A filter question asked whether the participant had a headache in the past 12 months. Subjects who answered affirmatively were asked to complete subsequent questions that addressed the International Classification of Headache Disorders – 3 ^rd^ edition (ICHD-3) criteria for migraine, TTH, and cluster headache: 29

- the number of attacks (categories: 1, 2 to 4, 5 to 9, or ≥10);
- duration (in minutes, hours, and days);
- location (unilateral or bilateral);
- quality (pulsating or pressing);
- intensity (mild, moderate, or severe);
- aggravation by routine physical activity;
- presence of nausea, vomiting, photophobia, and phonophobia;
- presence of aura and its characteristics;
- presence of autonomic symptoms and restlessness.

Premonitory and postdromic symptoms were surveyed by asking participants to select from a list in random order, those that occurred up to 48 hours before and after the headache phase, respectively. Possible answers for the premonitory phase were: phonophobia, lack of concentration, photophobia, neck pain, personality changes, mood changes, smell aversion, numbness, loss of appetite, fatigue, binge eating, unilateral rhinorrhea, unilateral lacrimation, or no symptom at all. Any additional symptoms could be reported in a text box. We analyzed the frequency of the six most common symptoms. The questionnaire was structured by a neurologist with training and experience in treating patients with headaches (EJP).

Study participants were divided into two groups: Mennonite Group (MG) and Control Group (CG). Subjects were included in the MG if they had direct Mennonite ancestry, either unilateral (only father or only mother) or bilateral (father and mother). Control group members could not have any Mennonite ancestry but should be a part of the Mennonite community, sharing daily customs and habits – most cases were spouses (husbands or wives of Mennonites).

All participants were older than 18 years old and suffered from headaches as defined by the ICHD-3. 29 We excluded the subjects who did not fully answer the questions about headaches.

A pilot test involved 38 community members, ensuring the effectiveness of the methodology, correcting doubts, and adding new information to the questionnaire. The final sample was composed of individuals from the urban Mennonite communities established in Curitiba: Primeira Igreja Irmãos Menonitas do Boqueirão, Igreja Irmãos Menonitas de São José dos Pinhais, Igreja Nova Aliança, and Igreja Irmãos Menonitas do Xaxim. Other groups were invited but did not participate in the study. The lack of adherence from other centers may have been related to the difficulty in making personal contact with them, which were far from the research location of the investigators.

The groups were compared concerning age, gender, type of headache, presence of aura, frequency of attacks, pain location and severity (using the numeric rating scale - NRS), attack duration, analgesic consumption, and characteristics of the premonitory and postdromic phases. When one of the diagnostic criteria for migraine was not met, we considered the case as probable migraine. 29

The results were summarized using the mean, standard deviation (SD), median, minimum, maximum, and frequency. Quantitative variables were compared using the student *t* -test for independent samples or the nonparametric Mann-Whitney test. Regarding categorical variables, comparisons were made using the Fisher exact test or the chi-squared test. P-values < 0.05 indicated statistical significance. Data were analyzed with the computer software IBM SPSS Statistics for Windows, v.28.0 (IBM Corp., Armonk, NY, USA).

## RESULTS

One-hundred and twenty individuals answered the questionnaire (54 from the CG and 66 from the MG). Of these, 17 returned incomplete forms and were excluded. Thus, the final analysis included 103 subjects (CG, *n* = 45; MG, *n* = 58) whose general characteristics are summarized in Table 1 . The groups did not differ concerning age or gender. The mean age of the participants was in the early 4 ^th^ decade of life. Most members were female, accounting for 73.3% of the CG and for 60.3% of the MG.

**Table 1:** General characteristics of the sample

Variable	Controls ( *n* = 45)	Mennonites ( *n* = 58)	*p-value*
Age (years old)	42.8 ± 11.7	41.3 ± 16.7	0.582
Female	33 (73.3%)	35 (60.3%)	0.337
Migraine	41 (91.1%)	47 (81.0%)	0.172
MO	34 (75.6%)	42 (72.4%)	0.719
Aura	20 (44.4%)	23 (39.7%)	0.689
Nonvisual aura	8 (17.8%)	8 (13.8%)	0.596
TTH	4 (8.9%)	9 (15.5%)	0.381
Pain intensity (NRS)	6.4 ± 2.4	6.2 ± 2.1	0.820
Frequency of headaches (days/month)			
< 1	9 (22.5%)	11 (20.8%)	0.821
1–3	21 (52.5%)	28 (52.8%)	
4–14	7 (17.5%)	12 (22.6%)	
>14	3 (7.5%)	2 (3.8%)	

Abbreviations: MO, migraine without aura; NRS, numeric rating scale; TTH, tension-type headache.

Note: All data are summarized as mean  ± standard deviation or count (relative frequency).

Migraine was the most common headache, found in 91.1% and in 81.0% of the CG and MG, respectively. Aura was a common finding (CG: 44.4%, MG: 39.7%), exceeding the prevalence of TTH (CG: 8.9%, MG: 15.5%). However, the distribution of the headache types did not differ between groups.

Most participants complained of moderate to severe pain occurring on 1 to 3 days per month. Chronic headache (> 14 days/month) was found in 7.5% of the CG and in 3.8% of the MG. The distribution of the severity and frequency of headaches did not show statistically significant differences between the two groups.

The migraine symptoms in the different phases were further evaluated. A premonitory phase was identified by 80.0% ( *n* = 36) and 81.0% ( *n* = 47) of the CG and MG, respectively ( *p* = 1.00). The postdromic phase was recognized by 28.9% ( *n* = 13) and 36.2% ( *n* = 21), respectively ( *p* = 0.528). However, the comparison by phase and symptoms did not differ between the two groups ( Table 2 ).

**Table 2:** Premonitory and postdromic symptoms

Variable		Controls ( *n* = 45)	Mennonites ( *n* = 58)	*p-value*
Premonitory	Phonophobia	20 (44.4%)	18 (31.0%)	0.217
	Inattention	13 (28.9%)	14 (24.1%)	0.654
	Photophobia	11 (24.4%)	15 (25.9%)	1
	Neck pain	12 (26.7%)	18 (31.0%)	0.668
	Personality change	16 (35.6%)	11 (19.0%)	0.072
	Mood changes	16 (35.6%)	11 (19.0%)	0.072
Postdromic	Sleep disorder	0 (0%)	1 (1.7%)	1
	Fatigue or demotivation	9 (20.0%)	13 (22.4%)	0.813
	Disorientation or inattention	2 (4.4%)	3 (5.2%)	1
	Irritability or bad mood	0 (0%)	4 (6.9%)	0.130
	Body pain	2 (4.4%)	0 (0%)	0.188

Regarding pain location, the CG was characterized by a higher frequency of retro-orbital pain (55.6%) than that found in the MG (32.8%; *p* = 0.027). The comparison between groups showed no other statistically significant difference concerning the location and laterality of pain ( Table 3 ). There was, however, a tendency toward a higher frequency of shoulder pain in the CG ( *p* = 0.055).

**Table 3:** Pain location

Variable	Controls ( *n* = 45)	Mennonites ( *n* = 58)	*p-value*
Unilateral fixed pain	7 (15.6%)	13 (22.4%)	0.457
Unilateral shifting pain	13 (28.9%)	13 (22.4%)	0.498
Bilateral pain	25 (55.6%)	32 (55.2%)	1.00
Frontal	24 (53.3%)	26 (44.8%)	0.431
Temporal	29 (64.4%)	36 (62.1%)	0.839
Parietal	5 (11.1%)	10 (17.2%)	0.416
Occipital	9 (20.0%)	13 (22.4%)	0.813
Retroorbital	25 (55.6%)	19 (32.8%)	0.027*
Neck	15 (33.3%)	19 (32.8%)	1.00
Shoulder	8 (17.8%)	3 (5.2%)	0.055
Masseter	2 (4.4%)	2 (3.5%)	1.00

Note: * *p* -value < 0.05.

**Figure 1:**
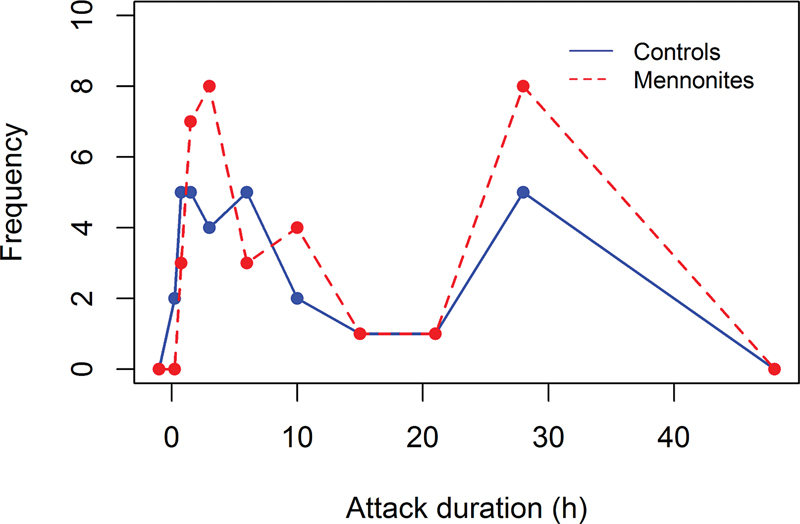
Frequency polygon of the duration of the headache attacks. There was no significant difference between the control (solid blue line) and Mennonite (dashed red line) groups ( *p* = 0.805).

The duration of the attacks of 38 subjects (CG: 15; MG: 23) was uncertain due to the use of analgesics in all episodes. Among the rest who knew how long their attacks lasted (63.1%), about half of them reported pain that persisted for < 4 hours (CG: *n* = 16, 53.3%; MG: *n* = 18, 51.4%). The duration of pain did not differ between groups ( *p* = 0.805; Figure 1 ).

In fact, most individuals used some form of acute treatment to relieve their headaches (CG: *n* = 37, 84.1%; MG: *n* = 45, 78.9%; *p* = 0.612). Nevertheless, simple analgesics were the most widely used class of drugs ( Table 4 ). Specific treatments for migraine, such as triptans (CG: 11.1%; MG: 17.2%) and ergot derivatives (CG: 4.4%; MG: 5.2%), were used less frequently. Headache management did not differ between groups.

**Table 4:** Analgesics used by the respondents

Analgesic	Controls ( *n* = 45)	Mennonites ( *n* = 58)	*p-value*
Dipyrone	23 (51.1%)	22 (37.9%)	0.230
Paracetamol	12 (26.7%)	15 (25.9%)	1.00
NSAIDs	12 (26.7%)	13 (22.4%)	0.649
Triptans	5 (11.1%)	10 (17.2%)	0.416
Ergotamine	2 (4.4%)	3 (5.2%)	1.00

Abbreviation: NSAIDs, non-steroidal anti-inflammatory drugs.

Regarding comorbidities, there were no differences in anxiety scores (GAD-7), depressive thinking distortions (EPD), disability due to headache (MIDAS), and intensity of dysautonomic symptoms (COMPASS 31) between the two groups ( Table 5 ). The CG, however, showed more severe depressive symptoms (PHQ-9) and sleepiness (ESS) than the MG.

**Table 5:** Scores for EPD, GAD-7, PHQ-9, ESS, MIDAS, and COMPASS 31 scales

Scale	Controls ( *n* = 45)	Mennonites ( *n* = 58)	*p-value*
EPD	70.8 ± 7.0	71.9 ± 6.1	0.406
GAD-7	9 (1–21)	5 (0–19)	0.092
PHQ-9	7 (0–22)	5 (0–24)	0.031*
ESS	6 (0–15)	4 (0–17)	0.048*
MIDAS	0 (0–270)	0 (0–108)	0.689
COMPASS 31	20.1 (0–52.7)	21.4 (0–51.0)	0.698

Abbreviations: COMPASS, Composite Autonomic Symptom Score; EPD, Depressive Thoughts Scale; ESS, Epworth Sleepiness Scale; GAD-7, General Anxiety Disorder-7; MIDAS, Migraine Disability Assessment; PHQ-9, Patient Health Questionnaire-9.

Notes: All data are summarized as mean ± standard deviation or median (minimum-maximum). * *p* -value < 0.05.

## DISCUSSION

The semiological aspects of individuals with headaches were similar between the two groups. Migraine accounted for a higher proportion of headache cases than expected in the general population. This finding is possibly due to the selection bias of the study respondents and the more significant disability caused by migraine. Still, the two groups showed a similar prevalence of MO, MA, and TTH, demonstrating that the Mennonite ancestry likely confers neither protective nor risk effects for these conditions, at least in the urban environment of Curitiba (Mennonites from rural communities were not assessed). These results shall still be appreciated with caution, considering the small sample size.

The primary aura subtype is characterized by visual symptoms lasting from 5 to 60 minutes and sometimes up to 24 hours (extended aura). We found a high prevalence of MA in both groups, possibly because of methodological selection bias. 15 Subjects suffering from MA often present with severe attacks associated with focal neurological deficits, which cause significant concern. Other epidemiological findings were consistent with a subpopulation of individuals with headaches, such as a high prevalence of females and a mean age in their early forties.

A key feature of the present study was the evaluation of the clinical characteristics of migraine in two populations of different ancestries under the influence of a shared urban environment. This design allows us to distinguish the genetic contribution to the manifestation of the disorder. For this purpose, all migraine stages (premonitory, aura, attack, and postdromic) were carefully evaluated. The premonitory phase is associated with hypothalamic activation, 30 which may precede the headache by up to 48 hours. Depending on the study methodology, the prevalence of premonitory manifestations ranges from 9% 31 to more realistic figures such as 87%. 14 The present study found values consistent with the latter. Since the study groups did not differ regarding the premonitory phase, it seems that Mennonites and non-Mennonite Brazilians sharing the urban environment did not differ regarding the onset of migraine.

The attack pattern (duration, intensity, and frequency) was similar in both groups. In Brazil, it is estimated that 9.6% of those with headaches have more than 14 days of pain per month, while the remaining 90.4% have an episodic course. 32 We found attack frequencies similar to those values. However, the duration of the attacks reported by our sample was shorter than expected, considering the high prevalence of migraine. This discordant finding is probably due to the retrospective nature of the study. Besides, about a third of the participants always used analgesics and could not estimate the duration of their attacks. It is reasonable to assume that this subgroup contained many who suffered from longer-lasting migraine episodes. There was no difference between groups regarding the type of analgesics being taken. We noticed a low frequency of use of more specific medications for migraine. Drugs such as triptans show greater efficacy in controlling the attacks, although their costs limit their use.

Migraine headache is usually felt over the frontotemporal region. 29 Not surprisingly, this site was the most affected by headaches in both groups. However, the CG complained of more frequent pain in the retroorbital region, an adjacent area. They also showed a tendency toward a higher proportion of pain over the shoulders, an extratrigeminal area. Together, these findings suggest a phenotype characterized by more diffuse pain in the CG. Our group showed that this fact might be associated with an alteration in sensory processing, and its co-occurrence with allodynia is common. 33

The postdromic phase of migraine is of extreme interest, as data in the literature is scarce. We evaluated and observed that they occur and are equally prevalent in both populations, affecting about one-third of their members. Manifestations of fatigue and demotivation were the most prevalent, demonstrating that the impact of this disease goes beyond pain symptoms.

The analysis of comorbidities demonstrated that the CG had more sleepiness and depressive symptoms than the MG. These variables were not sufficiently capable of influencing the migraine behavior in these groups. Regarding drowsiness, assessed using the ESS, it can occur as a prodrome, a manifestation in the interictal period, or a symptom of recovery. 14 16 Contradictory studies demonstrated that sleepiness could occur due to migraine itself, although it might also be a symptom of depression, anxiety, or other migraine comorbidity reducing sleep quality. 34

A strong correlation exists between migraine and depression, especially in high-frequency and chronic headaches. Buse et al. 35 used the PHQ-9 to study a sample in which a quarter of the 11,603 migraineurs had moderate to severe depression. There was an 87% increased risk of moderate to severe depression in those with high-frequency attacks (8 to 14 headache days per month) compared with participants with lower-frequency migraine (0 to 7 headache days per month). 35 In the present study, there were no statistical differences in the frequency of attacks between the CG and MG, suggesting that Mennonite ancestry did not play a role in the susceptibility to migraine-associated depressive symptoms, at least in the urban environment. However, this is an exploratory study, and the differences in the ESS and PHQ-9 scores might not be clinically significant (a two-point difference between the CG and MG medians). Still, the EPD scores were similar between groups.

To the best of our knowledge, this is the first study to investigate headaches in the Mennonite population. However, its results must be interpreted with caution because of several limitations mainly related to the research design. The Mennonite population is relatively isolated, making it challenging to approach a large number of participants. Boldt et al. 2 conducted a study involving 430 Mennonites and found worse self-rated health and higher depression and anxiety scores on the Beck Depression Inventory and Beck Anxiety Inventory. These were different scales, and there was extensive participation from the Mennonite population of Colônia Nova, who scored higher on the scales and were not included in the headache survey. 2

A selection method other than spontaneous participation, also including rural communities, is required to obtain more reliable results regarding prevalence. Gathering information about the familial pattern of MO and MA associated with genome sequencing would shed more light on the role of genetic variants in this population. Prospective studies using headache diaries and including a larger contingent of individuals would allow a better estimation of the migraine behavior in the Mennonite population. Currently, headache data are being collected on a larger group in a rural setting.

Headaches are a very prevalent group of diseases and, especially migraine, are under genetic influence. The Mennonite population has been in a context of reproductive isolation throughout its history, resulting in its distinctive epidemiology. Our results did not show major differences in the prevalence and clinical characterization of headaches between Mennonites and non-Mennonite Brazilians in the urban environment. However, the latter showed more diffuse pain, sleepiness, and depressive symptoms.
